# Very early onset of autoimmune thyroiditis in a toddler with severe hypothyroidism presentation: a case report

**DOI:** 10.1186/s13052-016-0270-7

**Published:** 2016-06-18

**Authors:** Pierluigi Marzuillo, Anna Grandone, Silverio Perrotta, Laura Ruggiero, Carlo Capristo, Caterina Luongo, Emanuele Miraglia del Giudice, Laura Perrone

**Affiliations:** Department of Woman, Child and General and Specialized Surgery, Seconda Università degli Studi di Napoli, Via L. De Crecchio n° 2, 80138 Naples, Italy

**Keywords:** Anaemia, Hypothyroidism, Creatine phosphokinase, Creatinine, Renal function, Case report

## Abstract

**Background:**

In infants under 3 years of age acquired primary hypothyroidism caused by autoimmune thyroiditis is very rare. Hypothyroidism can manifest with different signs and symptoms and has a wide range of presentations from subclinical hypothyroidism to overt form. We describe a child with acquired autoimmune thyroiditis during a very early period of life and with a severe hypothyroidism presentation.

**Case presentation:**

A 22-month-old white male patient with normal neonatal screening presented with a six-month history of asthenia and cutaneous pallor. At general clinical and biochemical exams he showed weight gain, statural growth deceleration, poor movements, sleepy expression, instability while walking, myxoedema, bradycardia, open anterior fontanelle, changes in the face habitus, macrocytic anaemia, ascites, and high CPK, creatinine and cholesterol levels. Acquired autoimmune thyroiditis was the final diagnosis. The thyroxine replacement therapy normalized all the clinical and biochemical abnormalities but at the age of 30 months his mental age showed a delay of 6 months.

**Conclusions:**

Our case could give useful learning points: i) although the screening for congenital hypothyroidism is routinely performed, a severe hypothyroidism (for example due to autoimmune thyroiditis) can anyway occur early in life and the clinicians should consider this possibility; ii) hypothyroidism can have a misleading and multi-face clinical presentation; iii) anemia, rhabdomyolysis and high creatinine levels should always include the hypothyroidism in the differential diagnosis; iv) thyroxine replacement therapy is able to revert all the clinical manifestations related to the hypothyroidism; v) evaluating the patient’s previous pictures could play an important role in resolving a diagnostic conundrum.

## Background

Acquired primary hypothyroidism in adults and adolescents is often caused by autoimmune thyroiditis [1]. In infants under 3 years of age acquired primary hypothyroidism caused by autoimmune thyroiditis is very rare [2–4]. Moreover, with the introduction of the neonatal screening the clinically evident hypothyroidism in the first years of life has become uncommon. Hypothyroidism can manifest with different signs and symptoms and has a wide range of presentations from subclinical hypothyroidism to overt form. After the introduction of the neonatal screening for congenital hypothyroidism, anemia, rhabdomyolysis and renal failure have been rarely reported in children as presenting symptoms of hypothyroidism, such as in adults [5–11]. We describe a very young child with acquired autoimmune thyroiditis and severe hypothyroidism presentation (including anemia, rhabdomyolysis and renal failure).

## Case presentation

**Fig. 1:**
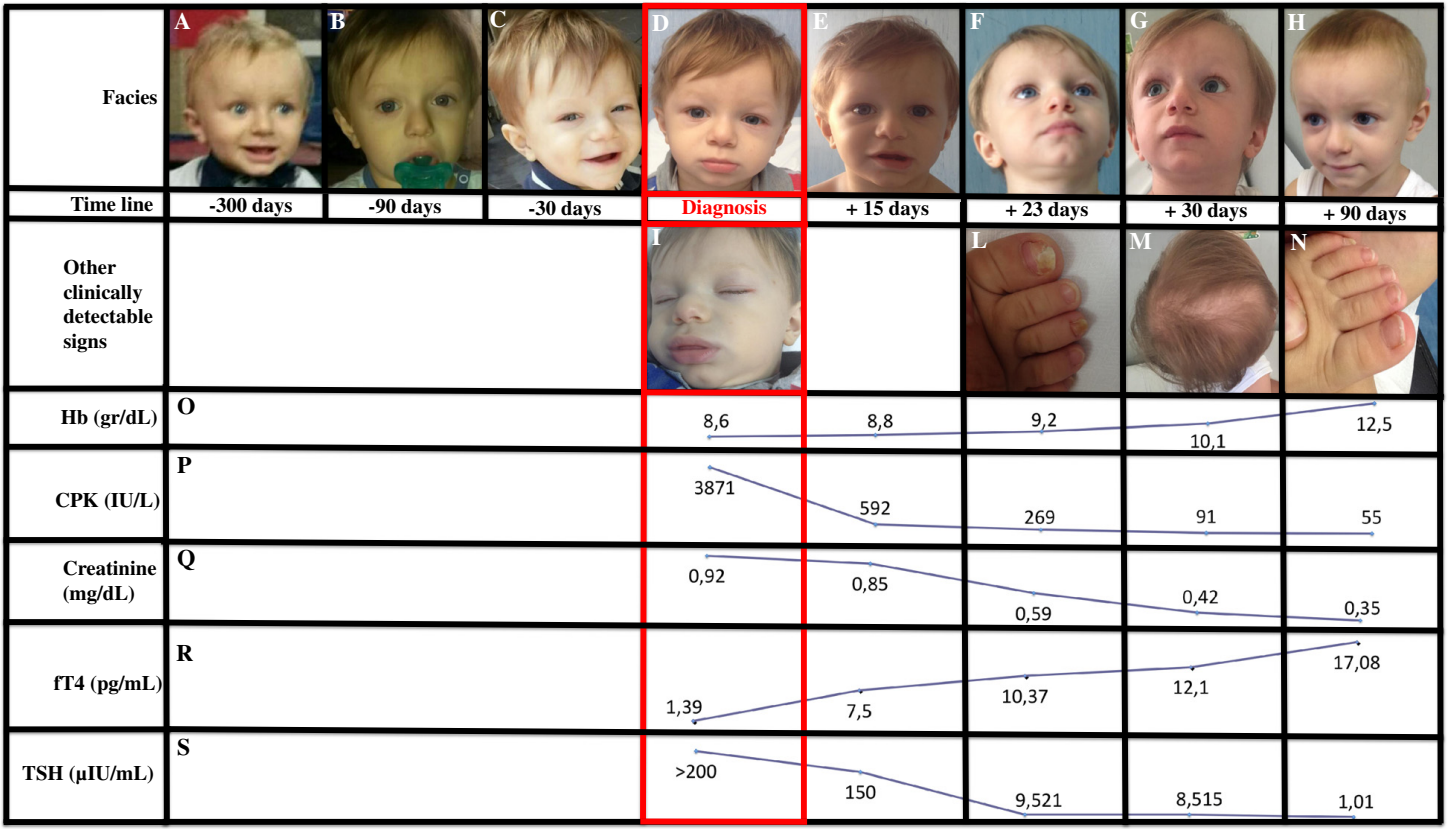
Child’s facial features and principal biochemical exams modifications. Panels A-D: child’s previous pictures and picture at diagnosis (before starting the therapy) demonstrating a modification of the facial features due to hypothyroidism. Panels E-H: child’s pictures after the treatment start. The child’s face changed again, returning to the child’s face before the onset of hypothyroidism. Panel L and N: ungual dystrophy and its recovery. Panel M: alopecia. Panels O-S: principal biochemical values at diagnosis and after the treatment start. From the beginning of the treatment, a gradual normalization of Hb, CPK, Creatinine, fT4 and TSH values was evident. The timeline indicates the moments in which the serum dosages were made, comparing them with the gradual changes in the facial habitus of the child

**Fig. 2:**
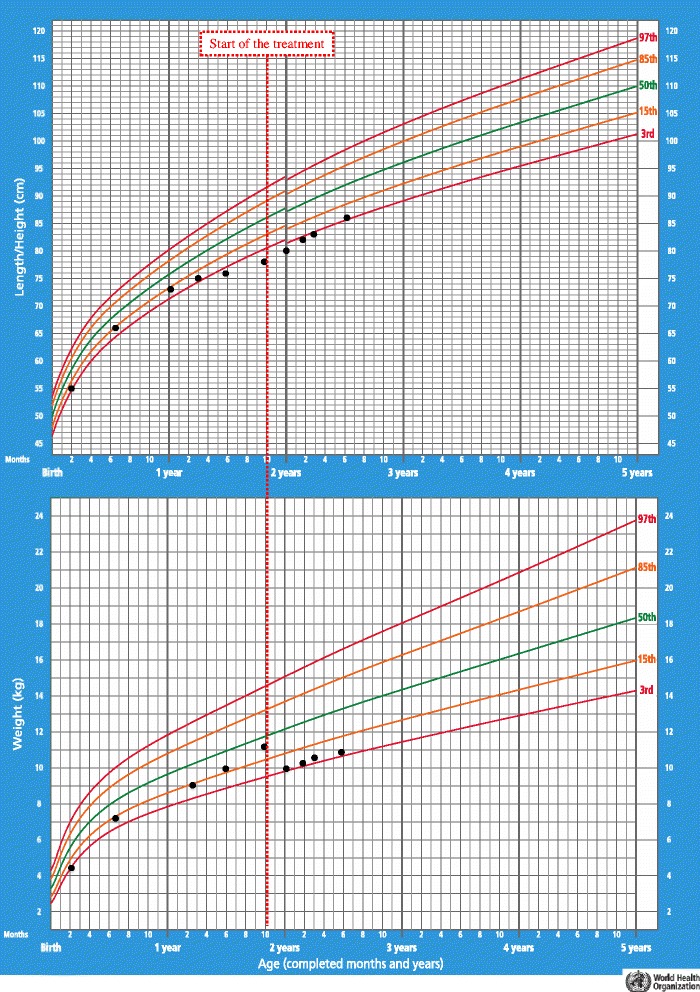
Child’s growth chart

A 22-month-old white male patient with unrelated parents was admitted to our Department for further investigation on a six-month history of asthenia and cutaneous pallor. Both pregnancy and delivery of the child were uneventful; the neonatal screening was normal. He had not yet started speaking. Physical examination revealed a pale child with poor movements and a sleepy expression (Fig. 1 Panel D), heart rate of 67 beats/minute, open anterior fontanelle and instability while walking. His thyroid gland was not palpable. The child’s weight and length were between 25^th^ and 50^th^ and below the 2^nd^ percentile, respectively. The weight-for-length percentile was 90–95^th^. The patient presented gradual statural growth deceleration with a concurrent weight gain during the last six months (Fig. 2). No history of diarrhoea, vomiting, or fever was present. No drug had been administered to the child. His mother was affected by autoimmune thyroiditis.

He had haemoglobin levels of 8.6 gr/dL (n.v. 11.2–14.2 gr/dL), a mean cell volume of 86.6 fl (n.v. 71–84 fl), and a mean cell haemoglobin concentration of 35.6 g/dL (n.v. 32–36 g/dL). The reticulocyte count was 0.0549 × 10^6^/L, and the leukocytes and platelets were within the normal range. General biochemical examinations showed high creatine phosphokinase (CPK) (3871 U/L) (n.v. <130 U/L), high lactate dehydrogenase (LDH) (1315 U/L) (n.v. 150–500 U/L) and high creatinine (0.92 mg/dL) (n.v. 0.03–0.50 mg/dL) levels. Urinalysis was normal. Our initial diagnostic suspects were congenital (transient erythroblastopenia of childhood, Diamond-Blackfan or Fanconi anaemia) or acquired (vitamin B12, folic acid deficiency) macrocytic anaemia (but the other symptoms were not justified), haemolytic anaemia (but jaundice was absent and the reticulocyte count was low), myositis/rhabdomyolysis or muscular dystrophy (this would not justify anaemia and high creatinine levels), coeliac disease (consistent with high CPK and LDH levels, but not with macrocytic anaemia and high creatinine levels), hypothyroidism (normal neonatal screening was in contrast with this hypothesis), and Systemic Lupus Erythaematosus (age at onset was atypical, the anemia was not autoimmune, but high LDH, CPK and creatinine levels would be consistent with this diagnosis). The following peripheral blood smear excluded the presence of atypical leukocytes. The reticulocyte count was confirmed low, and the haptoglobulin and bilirubin values were normal. The levels of vitamin B12, vitamin D and folic acid were normal. The abdomen ultrasound excluded an abdominal mass, but showed a small amount of free intra-peritoneal liquid. CPK isoforms were only of skeletal muscular origin, and the child’s parent presented normal CPK levels. Cholesterol serum levels were 250 mg/dL. Anti-TG2 antibodies were normal. IgM antibodies against Epstein-Barr virus, cytomegalovirus, adenovirus, parvovirus B19, toxoplasma were also absent, including antinuclear antibodies. Thyroid-stimulating hormone (TSH), free thyroxine (fT4), free triiodothyronin (fT3) were >200 μIU/mL, 1.39 pg/mL and 0.5 pg/mL, respectively. The levels of thyroid peroxidase antibodies and thyroglobulin antibodies were high (2017 IU/L and 1743 IU/L, respectively); sonographic thyroidal evaluation demonstrated normal anatomy with non-homogeneous echotexture. Because the neonatal screening for congenital hypothyroidism was normal, a diagnosis of hypothyroidism related to autoimmune thyroiditis was determined. On the basis of this diagnosis, we carefully re-evaluated the patient’s history and clinical examination, detecting additional signs and symptoms suggestive of hypothyroidism, which were previously unrecognized. We evaluated the child’s previous pictures and unexpectedly, a gradual change in the facial features was evident for 300 days preceding our observation (Fig. 1, Panels A-D). The heart rate (67 beats/minute) was not consistent with child anaemia. Moreover, we detected the presence of puffy tissues with apparent myxoedema (aided by a comparison of the child’s previous pictures) and the presence of macroglossia (evident during the child’s sleep) (Fig. 1, Panel I). The mental developmental index of the Bayley Scales of Infant Development showed a score of 62, his mental and motor development showed a delay of about 9 months. The bone age determined by TW2 method showed a delay of 12 months.

Levothyroxine treatment (5 μg/kg/day) was started and the dose was modulated to maintain TSH levels within the normal range (Fig. 1, Panel S). In the following 3 months, gradual normalization of Hb, MCV, CPK, creatinine, FT3, FT4 and cholesterol levels was shown (Fig. 1, Panels O-R); bradycardia and instability while walking disappeared within approximately 20 days and ascites after 1 month. The sleeping expression and paucity of movements were less evident after 15 days (Fig. 1, Panel E). Interestingly, after 23 days of treatment, the child initially showed ungual dystrophy (Fig. 1, Panel L) and then alopecia (Fig. 1, Panel M), resulting in the re-activation of nail and hair growth. At the age of 25 months, myxedema (and macroglossia) completely disappeared, as did alopecia and ungual dystrophy (Fig. 1, Panel N). An adequate weight-for-length percentile (25–50^th^) was achieved (Fig. 2). The anterior fontanelle was still open, and the child still has not begun to speak. The child’s face changed again, returning to the child’s face before the onset of hypothyroidism (Fig. 1, Panels D-H). Moreover, the child showed normal activity and interaction with the environment.

At the last follow up visit at the age of 30 months, his motor development and mental age progressed and were about 6 months behind his chronological age. With regards to language, he did not learn any words, but he was able to understand the orders and to accomplish them.

## Conclusions

Hypothyroidism in neonates and very young infants is usually caused by thyroid dysgenesis (associated with an absent, ectopic, or hypoplastic gland) or by thyroid hormones dyshormonogenesis defects [3]. The neonatal screening is able to detect this condition before it becomes clinically evident. Usually primary hypothyroidism in infancy is attributed to a failure of newborn screening to detect congenital hypothyroidism [2]. However, in young children hypothyroidism could be caused by chronic autoimmune thyroiditis [1–5], also if it is rare before the age of three years and can be expression of a constellation of polyglandular autoimmune endocrine deficiency syndromes [3, 5]. The peculiarity of our case report was the early onset of autoimmune thyroiditis with severe phenotype of hypothyroidism and an ill appearance of the patient (Fig. 1). Indeed, this child showed weight gain, statural growth deceleration, poor movements, sleepy expression, instability while walking, myxoedema, bradycardia, open anterior fontanelle, changes in the face habitus, macrocytic anaemia, ascites, and high CPK, creatinine and cholesterol levels. This kind of hypothyroidism presentation has become unusual after the introduction of the neonatal screening for congenital hypothyroidism; but with this case report we want underline that although the screening is routinely performed a severe hypothyroidism (for example due to autoimmune thyroiditis) can be anyway possible early in life.

However, the age at presentation of hypothyroidism (presumably after one year of age) and the normal neonatal screening made hypothyroidism an unlikely principal diagnostic hypothesis. Moreover, the important multi-organ involvement and ill appearance of the patient initially attracted our attention to life-threatening conditions.

We want underline the association between hypothyroidism and anemia. Hypothyroidism may lead to macrocytic anemia because of decreased bone marrow activity and decrease in erythropoietin secretion [10]. Very recently, it has been demonstrated that the prevalence of anemia was higher in overt hypothyroidism population compared with euthyroid group [8]. No similar studies are available in childhood but considering our case report and the other cases available in literature [9], the possibility exists that a similar trend could be present also in childhood.

The presence of impaired renal function in our patient resolving when the thyroidal function had been restored is also interesting. Another case resolving renal function with thyroxine replacement therapy is available in literature [11]. The pathophysiological mechanism is intriguing; the hypercreatinemia is related to the hypodynamic state that occurs in hypothyroidism, leading to a reduced glomerular filtration rate [11].

Hypothyroidism presents rarely with rhabdomyolysis in adults [6, 7] and very rarely in infants [5], and elevated levels of CPK can be seen in congenital hypothyroidism [12, 13]. The pathophysiological mechanism through which the hypothyroidism could lead to rhabdomyolysis is unknown. Probably, in our patient, the concomitant renal impairment favored the increase of CPK levels. Interestingly, the CPK levels rapidly decreased with the thyroxine replacement therapy.

As in the case described by Joergensen et al. [4], our patient presented impaired developmental outcomes, at the age of 30 months the mental development was partially recovered, but unfortunately the follow up period is too short to give more precise information about the outcomes.

The thyroiditis, in our child, started in a period of life in whom the thyroidal hormones are fundamental for a correct mental development [4], therefore a strict follow up and further future cognitive evaluations are needed to better define the outcomes and start proper interventions.

In conclusion, i) although the screening for congenital hypothyroidism is routinely performed, a severe hypothyroidism (for example due to autoimmune thyroiditis) can anyway occur early in life and the clinicians should consider this possibility; ii) hypothyroidism can have a misleading and multi-face clinical presentation. Thus, clinicians should always evaluate thyroid function when taking care of an infant with multi-organ involvement and normal neonatal screening; iii) anemia, rhabdomyolysis and high creatinine levels should always include the hypothyroidism in the differential diagnosis; iv) thyroxine replacement therapy is able to revert all the clinical manifestations related to the hypothyroidism; v) evaluating the patient’s previous pictures could play an important role in resolving a diagnostic conundrum.

## Abbreviations

CPK, high creatine phosphokinase; LDH, lactate dehydrogenase; TSH, thyroid-stimulating hormone; fT4, free thyroxine; fT3, free triiodothyronin
